# A randomised controlled trial of the effect of providing online risk information and lifestyle advice for the most common preventable cancers

**DOI:** 10.1016/j.ypmed.2020.106154

**Published:** 2020-09

**Authors:** Golnessa Masson, Katie Mills, Simon J. Griffin, Stephen J. Sharp, William M.P. Klein, Stephen Sutton, Juliet A. Usher-Smith

**Affiliations:** a The Primary Care Unit, Department of Public Health and Primary Care, University of Cambridge School of Clinical Medicine, Box 113, Cambridge Biomedical Campus, Cambridge CB2 0SR, UK; b MRC Epidemiology Unit, University of Cambridge, Institute of Metabolic Science, Cambridge CB2 0QQ, UK; c National Cancer Institute, Rockville, MD, USA

**Keywords:** Risk, Cancer, Risk perception, Behaviour, Communication, Randomised controlled trial

## Abstract

Few trial data are available concerning the impact of personalised cancer risk information on behaviour. This study assessed the short-term effects of providing personalised cancer risk information on cancer risk beliefs and self-reported behaviour. We randomised 1018 participants, recruited through the online platform Prolific, to either a control group receiving cancer-specific lifestyle advice or one of three intervention groups receiving their computed 10-year risk of developing one of the five most common preventable cancers either as a bar chart, a pictograph or a qualitative scale alongside the same lifestyle advice. The primary outcome was change from baseline in computed risk relative to an individual with a recommended lifestyle (RRI)^1^ at three months. Secondary outcomes included: health-related behaviours, risk perception, anxiety, worry, intention to change behaviour, and a newly defined concept, risk conviction. After three months there were no between-group differences in change in RRI (*p* = 0.71). At immediate follow-up, accuracy of absolute risk perception (*p* < 0.001), absolute and comparative risk conviction (*p* < 0.001) and intention to increase fruit and vegetables (*p* = 0.026) and decrease processed meat (*p* = 0.033) were higher in all intervention groups relative to the control group. The increases in accuracy and conviction were only seen in individuals with high numeracy and low baseline conviction, respectively. These findings suggest that personalised cancer risk information alongside lifestyle advice can increase short-term risk accuracy and conviction without increasing worry or anxiety but has little impact on health-related behaviour.

**Trial registration**: ISRCTN17450583. Registered 30 January 2018.

## Highlights

• Cancer risk information had no effect on computed risk or self-reported behaviour.
• There was also no effect on anxiety or cancer related worry.
• Accuracy and conviction of absolute risk perception increased in the short-term.
• Numeracy and baseline conviction moderate these changes.

## 1 Introduction

Approximately 40% of cancer cases can be attributed to lifestyle factors such as smoking, alcohol intake, diet, physical activity levels and sun protection (Parkin et al., 2011). Encouraging individuals to adopt healthy lifestyles offers a potentially cost-effective, long-term approach to cancer control.

**Fig. 1:**
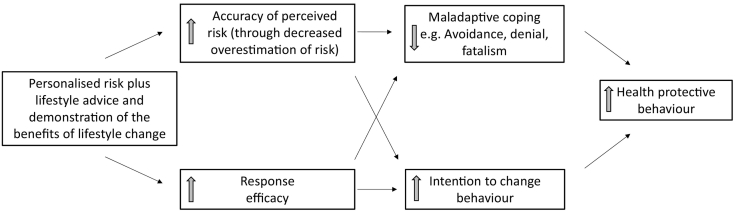
Proposed theoretical mechanism for intervention.

One strategy for targeting individuals is providing personalised information about risk. While evidence for behaviour change in other disease areas following the provision of personalised risk information is limited (Usher-Smith et al., 2015), the impact of cancer risk provision on behaviour is not known (Usher-Smith et al., 2018a), and there are reasons to believe that the impact of provision of information about risk of cancer might differ. People are more concerned about developing and dying from cancer than from cardiovascular disease (Scheideler et al., 2017; Klein et al., 2014) and a high proportion of individuals incorrectly believe their cancer risk to be higher than current estimates. There is also a lack of awareness of the role lifestyle plays in the development of cancer (*World Cancer Day: Half Don't Know About Link Between Diet and Cancer*, 2014) and a significant minority of the public continue to believe that cancer risk is unmodifiable (Ryan et al., 2015). Although originally developed to understand responses to fear appeals, two widely used theories of behaviour change (Rogers, 1975; Witte, 1992), can help describe how such overestimation and lack of response efficacy, or the perceived effectiveness of recommended actions, might be associated with maladaptive responses such as feelings of fatalism or hopelessness (McCaul and O'Donnell, 1998; Weinstein et al., 2004a; Waters et al., 2011) and decreased motivation to change behaviour. For example, individuals who overestimate their risk of developing cancer and are unaware of the benefits of changes in their lifestyle, may feel it is almost useless to try to stay healthy and so have low motivation to change. When accompanied by information about these risk factors and the benefits of change, personalised cancer risk information may therefore improve the accuracy of risk perception and reduce overestimation and increase response efficacy, in turn reducing maladaptive responses and motivating behaviour change (Fig. 1).

An important element of risk communication that influences accuracy of risk perception (hereafter referred to as risk accuracy) and may also have an impact on other risk beliefs and subsequent behaviour change is the format in which risk is presented (Lipkus, 2007; Hussein et al., 2008; Radcliffe and Klein, 2002; Trevena et al., 2013).

Risk conviction, a newly defined concept which represents “the subjective sense that one knows what one's risk belief is and confidence that this risk belief is accurate” (Taber and Klein, 2016), may also influence subsequent behaviour change by influencing the relationship between risk perception and behaviour. Previous research has shown that uncertainty about perceived risk is associated with lower odds of change in self-report dietary and physical activity measures (Orom et al., 2017a; Orom et al., 2017b) and that attitudes held with greater certainty are more stable and persistent and may have a stronger influence on behaviour (Glasman and Albarracín, 2006). However, no studies have measured risk conviction after provision of risk information and it is not known whether risk conviction can be altered.

In a parallel-group randomised controlled trial we therefore aimed to assess the short-term effects on health-related behaviours and risk beliefs, including risk perception and risk conviction, of three different formats (a bar chart, pictograph and qualitative scale) of personalised cancer risk information alongside lifestyle advice. We hypothesised that there would be a reduction in computed relative risk in the three risk groups combined when compared with the control group, there would be no difference between the two numerical presentations (bar chart and pictographs) and there would be greater reductions in computed relative risk in the qualitative group than the groups presented with numerical information. We further hypothesised that there would be increases in absolute and comparative risk accuracy and conviction in the three risk groups combined compared with the control group and greater increases in absolute accuracy and conviction in the numerical groups than the qualitative group.

## 2 Methods

The study is reported in accordance with the CONSORT guidelines (Schulz et al., 2010). Full details of the design and methods of the study are published elsewhere (Usher-smith et al., 2018b). Briefly, this was a web-based parallel-group, open randomised control trial with a control group that received cancer-specific lifestyle advice and three intervention groups that received their computed 10-year risk of developing cancer in one of three different formats alongside the same lifestyle advice.

### 2.1 Recruitment

1018 participants were recruited between 10 and 12 April 2018 using Prolific Academic (https://www.prolific.ac/). Eligible individuals were men and women aged 30–74 years, resident in the UK, without a past history of cancer and with a Prolific approval rating (reflecting the proportion of studies completed to the standard accepted by researchers) ≥ 95% (Peer et al., 2014). Participants received a small financial reward (£3) for taking part. Participants were randomised 1:1:1:1 to four groups at an individual level based on computer generated random numbers within block sizes of eight. Randomisation was stratified by sex, risk relative to an individual with a recommended lifestyle (≤ or >1.5) and age (≤ or >40 years).

To identify inattentive participants and increase the reliability and validity of responses (Oppenheimer et al., 2009), an instructional manipulation check (“*It is important that you pay attention in this study. Please tick ‘strongly disagree’*”) was included in the baseline questionnaire. Participants who failed this check or who failed to provide sufficient information for the calculation of a risk estimate were excluded prior to randomisation.

### 2.2 Computed risk and the interventions

**Fig. 2:**
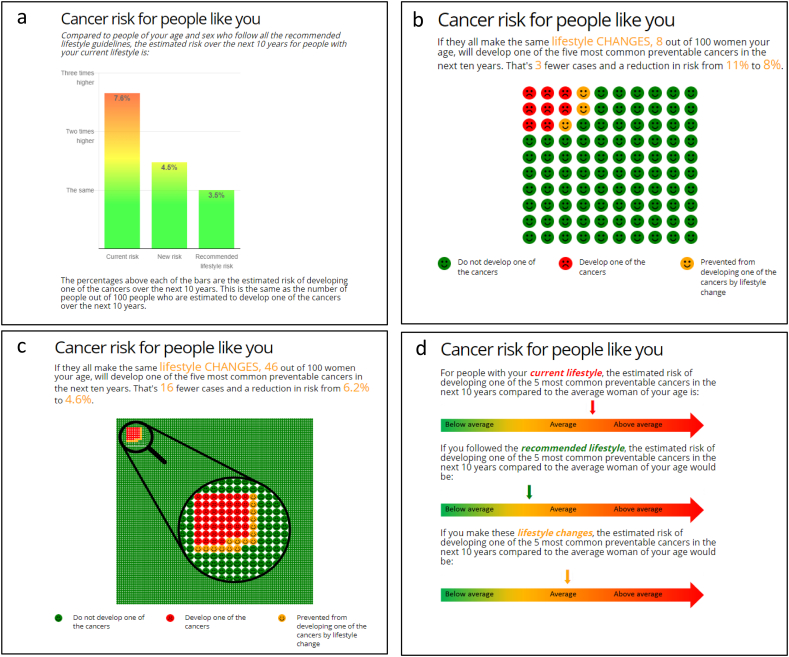
Risk presentation formats. (a) Bar chart; (b) 100 icon pictograph for those with an absolute risk >8%; (c) 1000 icon pictograph with a magnified section of 100 icons for those with a relative risk ≤8%; (d) qualitative scale.

Reproduced with permission from Usher-Smith JA et al. BMC Public Health 18(1):796. doi: https://doi.org/10.1186/s12889-018-5712-2.

It was not possible to blind participants to which intervention they received. Participants in all groups were provided with lifestyle advice on six target behaviours: smoking cessation, weight loss, reduction of alcohol consumption, increasing fruit and vegetable consumption, decreasing red and processed meat consumption and increasing physical activity. The three intervention groups additionally saw their computed 10-year risk of developing one of the five commonest preventable cancers (lung, colorectal, bladder, kidney and oesophageal for men and breast, lung, colorectal, endometrial and kidney for women). This risk was computed using a lifestyle-based risk score developed for this purpose (Usher-Smith et al., 2019). In brief, lifestyle factors for each cancer were selected from the European Code against Cancer and estimates of the relative risks from meta-analyses of observational studies. Average population values for each risk factor were then used to calculate estimates of 10-year risk of developing one or more of the given cancers. In men the risk model showed good discrimination (AUC: 0.71, 95% CI 0.69–0.73) and calibration. Discrimination was lower in women (AUC: 0.59, 0.57–0.61) but calibration was good. Participants were presented with the estimated 10-year risk in one of the three formats (Fig. 2). Further information about the risk scores was also provided (Supplementary file 1) and the participant information sheet included details of organisations for further information and support (Supplementary file 2). Participants in all three groups were able to set target values for each of the target behaviours and see the effect of this on their risk.

### 2.3 Measures

To enable us to assess the impact on behaviour change independent of age and sex, the primary outcome was change from baseline to three months in computed risk relative to an individual of the same age and sex with a recommended lifestyle (RRI). Full details of the secondary outcomes are in the published protocol (Usher-smith et al., 2018b) and Supplementary Table S1.

### 2.4 Statistical analyses

All analyses were performed based on the intent-to-treat principle.

#### 2.4.1 Primary outcome

We used analysis of covariance to calculate change in RRI at three-month follow-up. Any difference between the four randomised groups was tested using an F test, followed by estimation of four pairwise contrasts between the study groups: 1) Control group vs the three risk groups combined; 2) bar chart presentation vs pictographs; 3) bar chart presentation vs qualitative scale; and 4) pictographs vs qualitative scale. An F test was used to test for interactions between the groups and: age (≤40/>40 years), sex (to assess for differences due to variable effects of lifestyle change across the cancers included in the sex specific risk models), baseline RRI (≤1.5/>1.5), self-perceived risk at baseline below or above computed risk and numeracy (<2/≥2). Analyses were repeated within the subgroups where the *p*-value for interaction was <0.05.

#### 2.4.2 Secondary outcomes, acceptability and process measures

We used the same approach for continuous secondary outcome variables (awareness of cancer risk factors, risk conviction, maladaptive coping, anxiety, cancer worry, BMI, physical activity and diet and alcohol). Logistic regression (adjusted for baseline) was used to analyse binary secondary outcome variables (absolute and comparative risk accuracy and smoking status). Absolute risk accuracy was defined as a difference of 5% or less between computed and perceived absolute risk, with sensitivity analyses using ±1% and ±10% limits performed due to the heterogeneity of the definition of risk accuracy in the literature (Hussein et al., 2008; Radcliffe and Klein, 2002; Silarova et al., 2017). Comparative risk accuracy was defined as agreement between computed and perceived comparative risk on a continuous seven-point scale from “Much lower” to “Much higher”. A sensitivity analysis defining average comparative risk perception as responses between 3 and 5 on the scale was also performed. Linear regression was used to analyse intention to change behaviour, self-efficacy and response efficacy (all considered as continuous variables) because these outcomes were measured only at immediate follow up. As there was high correlation (r > 0.8) between the two questions used to measure risk conviction, the responses to both questions was combined for all analyses.

As risk perception has been demonstrated to be influenced by age, sex, numeracy and whether individuals over or underestimate their risk at baseline (Silarova et al., 2017; Wang et al., 2012; Reyna et al., 2010), we also planned a priori to explore the impact of these factors on risk accuracy and risk conviction. We did this by including age (<40 years/≥40 years), sex, numeracy (<2/≥2), baseline risk accuracy (underestimate/accurate/overestimate) and baseline risk conviction (<4/≥4) as categorical interaction terms within the logistic regression or ANCOVA models detailed above. Where there was evidence against the null (*p* < 0.05) for any of these interactions the variables are summarised separately.

Confidence intervals (98.75%, based on a Bonferroni corrected significance threshold of 1.25%) are presented for all outcomes to acknowledge that four pairwise comparisons were performed. All analyses were performed using STATA V.15.1.

#### 2.4.3 Sample size

Based on the mean (1.77) and standard deviation (0.97) of the computed risk of developing one or more of the five chosen cancers relative to an individual with a recommended lifestyle in the EPIC-Norfolk cohort used for external validation of the risk scores (Day et al., 1999, Usher-Smith et al., 2019), and assuming a 10% loss to follow-up, we estimated that with 1000 participants (250 per group) we could detect a baseline-adjusted between-group difference of 0.3, with 98.75% confidence and 79–83% power (depending on the degree of correlation between baseline and follow up values).

#### 2.4.4 Post-hoc analyses

To investigate the impact of provision of risk information amongst those who engaged with the lifestyle advice, we performed post-hoc analyses including only those participants who had viewed one or more of the lifestyle pages.

## 3 Results

### 3.1 Participants

**Fig. 3:**
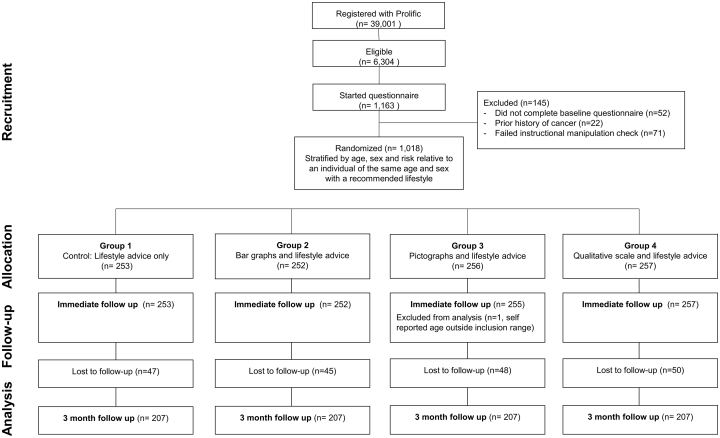
Consort flow diagram.

1163 individuals completed the online consent form. Of these, 145 were excluded prior to randomisation. 863 of the 1018 who were randomised completed the three-month follow up questionnaire (85% retention) (Fig. 3). Table 1 summarises the baseline characteristics of participants in each group. Characteristics were similar across the groups. The mean age of participants was 43.9 years (SD 10.0). 49% were aged between 30 and 40 years. Females made up 71% of the cohort and 93% were White Caucasian.

**Table 1:** Baseline characteristics of participants.

	Group 1: Lifestyle advice (N = 253)		Group 2: Bar charts (N = 252)		Group 3: Pictographs (N = 255)		Group 4: Qualitative (N = 257)	
	*n*	Mean (SD)/% (*n*)	*n*	Mean (SD)/% (*n*)	*n*	Mean (SD)/% (*n*)	*n*	Mean (SD)/% (*n*)
Age (years)	253	42.4 (9.0)	252	43.4 (10.4)	255	42.7 (10.0)	257	43.0 (10.4)
Age group (years)	253		252		255		257	
30–40		49.8 (126)		48.4 (122)		48.2 (123)		49.8 (128)
41–50		28.9 (73)		26.2 (66)		27.1 (69)		27.6 (71)
>50		21.3 (54)		25.4 (64)		24.7 (63)		22.6 (58)
Sex (% female)	253	71.2 (180)	252	71.8 (181)	255	71.0 (181)	257	71.6 (184)
Ethnicity (% white)	253	94.5 (239)	250	92.8 (232)	254	92.9 (236)	255	93.3 (238)
Family history cancer (% yes)	251	35.5 (89)	249	35.3 (88)	253	38.3 (97)	253	40.7 (103)
Estimated risk^a^								
Absolute (%)^b^	253	0.8 (0.5–2.5)	252	0.8 (0.5–3.1)	255	0.8 (0.5–2.7)	257	0.9 (0.5–2.5)
Relative to individual of same age and sex^b^	253	1.0 (0.9–1.2)	252	1.0 (0.9–1.2)	255	1.0 (0.8–1.2)	257	1.0 (0.8–1.2)
Relative to individual of the same age and sex with a recommended lifestyle^b^	253	1.3 (1.0–1.6)	252	1.3 (1.0–1.6)	255	1.2 (1.0–1.6)	257	1.2 (1.0–1.6)
Risk perception								
Absolute (%)^b^	223	25 (10–50)	227	20 (10–50)	224	25 (10–50)	236	30 (10–50)
Comparative (relative to individual of the same age and sex) (range 1–7)^b^	253	4 (3–5)	252	4 (3–5)	255	4 (3–4)	257	4 (3–5)
Absolute risk accuracy	223		227		224		236	
Underestimate		0.5 (1)		0		0.5 (1)		0
Accurate		18.8 (42)		20.7 (47)		18.8 (42)		17.8 (42)
Overestimate		80.7 (180)		79.3 (180)		80.8 (181)		82.2 (194)
Comparative risk accuracy	253		252		255		257	
Underestimate		41.1 (104)		34.5 (87)		38.4 (98)		37.0 (95)
Accurate		37.2 (94)		39.7 (100)		42.8 (109)		41.3 (106)
Overestimate		21.7 (55)		25.8 (65)		18.8 (48)		21.8 (56)
Risk conviction (range 0–7)								
Absolute^b^	246	3.0 (1.5–4.0)	250	3.0 (1.5–4.5)	252	2.5 (1.5–4.0)	256	2.5 (1.5–4.0)
Comparative^b^	243	3.0 (2.0–4.5)	245	3.0 (2.0–5.0)	248	3.0 (2.0–4.0)	245	3.0 (2.0–4.0)
Numeracy	253		251		254		255	
High (≥2)		72.7 (184)		73.3 (184)		74.4 (189)		75.3 (192)
Low (<2)		27.3 (69)		26.7 (67)		25.6 (65)		24.7 (63)
Concern about developing cancer (range 0–7)^b^	249	5 (3–6)	250	5 (3–6)	252	5 (3–6)	257	5 (3–6)

a All risk estimates represent the 10 year risk of developing one of the five most common, gender specific, preventable cancers.

b Median (IQR).

The median perceived absolute risk across the four groups was 25% (IQR 10–50%). The median computed absolute risk of the study population was only 0.83% (IQR 0.53–2.63%). The vast majority of participants (81%) were therefore overestimating their risk at baseline. This included almost a fifth of participants who reported perceived absolute cancer risk of 50% at baseline (18%) and at immediate (16%) and three-month (21%) follow up. The median concern about developing cancer was 5 (IQR 3–6), with over half (52%) “Moderately”, “Very” or “Extremely” concerned. The median computed risk relative to an average individual of the same age and sex (RR) was 1 (IQR 0.8–1.2) and the median computed risk relative to an individual of the same age and sex with a recommended lifestyle (RRI) was 1.2 (IQR 1–1.6).

Median baseline absolute and comparative risk conviction were 2.5 (IQR 1.5–4) and 3 (IQR 2–4.5) respectively across the study groups. The majority of participants (>75%) reported baseline conviction scores at or below 4 (the midpoint of the scale), suggesting relatively low certainty and confidence in their risk perceptions.

### 3.2 Primary outcome

**Fig. 4:**
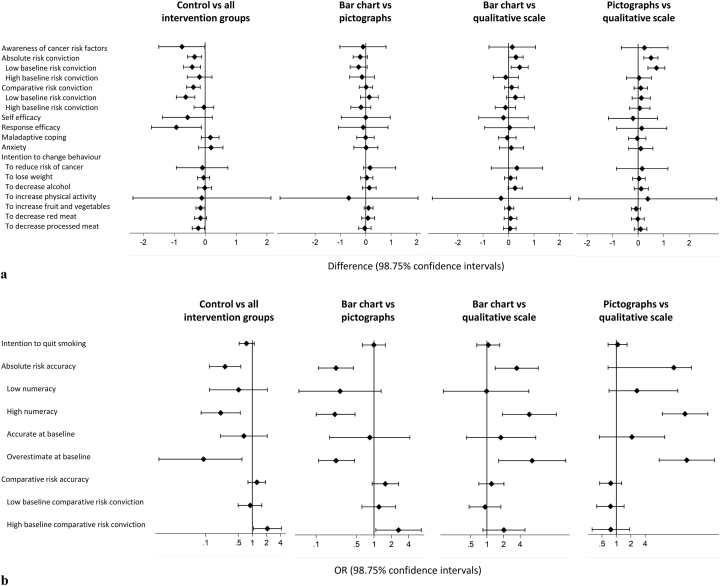
Pairwise analyses results for a) each continuous secondary outcome measure and b) each categorical secondary outcome measure at immediate follow up.

**Fig. 5:**
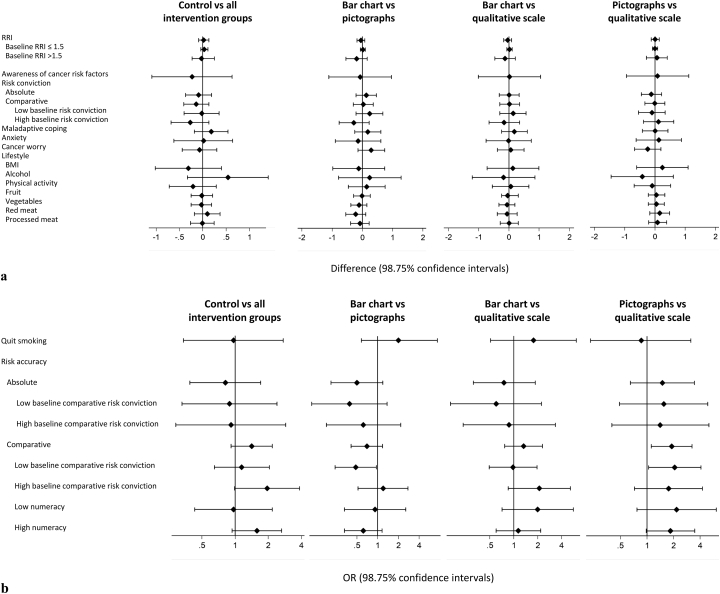
Pairwise analyses results for a) each continuous secondary outcome measure and b) each categorical secondary outcome measure at 3 month follow up. RRI – risk relative to an individual of the same age and sex with a recommended lifestyle.

Table 2, Table 3 show means and SDs for primary and secondary outcomes at baseline and change from baseline. Details of the four pairwise comparisons are presented in Fig. 4, Fig. 5 and in Supplementary Tables S2 and S3.

**Table 2:** Means and SDs of baseline and change from baseline to immediate follow-up values.

	Group 1: Lifestyle advice			Group 2: Bar charts			Group 3: Pictographs			Group 4: Qualitative		
	Baseline		Change^a^ from baseline	Baseline		Change^a^ from baseline	Baseline		Change^a^ from baseline	Baseline		Change^a^ from baseline
	n	Mean (SD)	Mean (SD)	n	Mean (SD)	Mean (SD)	n	Mean (SD)	Mean (SD)	n	Mean (SD)	Mean (SD)
*Secondary continuous outcomes*												
Awareness of cancer risk factors	238	40.0 (5.6)	2.9 (4.2)	241	40.9 (5.0)	3.4 (3.9)	237	40.3 (5.7)	3.7 (4.3)	242	40.7 (5.6)	3.3 (4.3)
Risk conviction												
Absolute	247	2.9 (1.5)	0.16 (1.1)	250	3.0 (1.7)	0.48 (1.5)	253	2.9 (1.6)	0.74 (1.6)	256	2.9 (1.5)	0.26 (1.1)
Comparative	243	3.2 (1.5)	−0.01 (1.0)	245	3.3 (1.6)	0.38 (1.4)	249	3.2 (1.5)	0.40 (1.3)	245	3.2 (1.5)	0.32 (1.3)
Self-efficacy	229	–	21.6 (4.6)	236	–	22.1 (4.3)	242	–	22.1 (4.3)	246	–	22.3 (4.1)
Response efficacy	229	–	22.9 (4.0)	240	–	23.8 (4.1)	236	–	24.0 (4.3)	233	–	23.8 (4.5)
Maladaptive coping	250	9.2 (2.1)	−0.12 (1.6)	249	9.3 (2.0)	−0.32 (1.6)	255	9.4 (2.0)	−0.35 (1.6)	256	9.2 (2.1)	−0.26 (1.5)
Anxiety (SF-SSAI)	242	12.0 (4.2)	0.37 (2.0)	245	12.2 (4.4)	0.21 (2.3)	252	12.3 (4.3)	0.17 (2.1)	246	12.0 (3.9)	0.13 (1.9)
Intention to change behaviour												
General	238	–	21.9 (4.2)	241	–	22.1 (4.6)	243	–	22.0 (4.6)	246	–	21.8 (4.5)
Weight	214	–	3.9 (1.0)	218	–	4.0 (1.0)	212	–	3.9 (1.0)	216	–	3.9 (1.0)
Alcohol	183	–	3.6 (1.1)	182	–	3.7 (1.1)	198	–	3.6 (1.0)	173	–	3.4 (1.1)
Physical activity	242	–	5.4 (12.2)	244	–	5.1 (10.5)	249	–	5.8 (13.4)	251	–	5.4 (12.0)
Fruit and veg	240	–	3.8 (0.8)	241	–	4.0 (0.8)	247	–	3.9 (0.8)	244	–	4.0 (0.7)
Red meat	208	–	3.2 (1.0)	204	–	3.4 (1.1)	214	–	3.3 (1.1)	206	–	3.4 (1.0)
Processed meat	209	–	3.5 (1.0)	211	–	3.7 (1.1)	206	–	3.7 (1.0)	206	–	3.7 (1.0)
Quit smoking	68	–	3.5 (1.0)	60	–	3.8 (1.1)	73	–	3.8 (1.0)	65	–	3.7 (1.1)


	Group 1: Lifestyle advice			Group 2: Bar charts			Group 3: Pictographs			Group 4: Qualitative		
	Baseline		Change^a^ from baseline	Baseline		Change^a^ from baseline	Baseline		Change^a^ from baseline	Baseline		Change^a^ from baseline
	n	% (n)	%	n	% (n)	%	n	% (n)	%	n	% (n)	%

*Secondary categorical outcomes*												
Risk accuracy												
Absolute	223	19 (42)	−3.0	227	21 (47)	6.5	225	19 (42)	30.1	236	18 (42)	−2.4
Low numeracy	62	18 (11)	−3.6	59	17 (10)	−1.9	57	26 (15)	10.4	56	16 (9)	0
High numeracy	161	19 (31)	−2.8	167	22 (37)	9.7	168	16 (27)	36.0	178	19 (33)	−3.1
Comparative	253	37 (94)	1.7	252	40 (100)	0.4	256	43 (110)	−10.8	257	41 (106)	−4.8

a Change from baseline was calculated as follow-up minus baseline. Immediate follow-up values given for those variables not measured at baseline.

**Table 3:** Means and SDs of baseline and change from baseline to 3 month follow-up values.

	Group 1: Lifestyle advice			Group 2: Bar charts			Group 3: Pictographs			Group 4: Qualitative		
	Baseline		Change^a^ from baseline	Baseline		Change^a^ from baseline	Baseline		Change^a^ from baseline	Baseline		Change^a^ from baseline
	n	Mean (SD)	Mean (SD)	n	Mean (SD)	Mean (SD)	n	Mean (SD)	Mean (SD)	n	Mean (SD)	Mean (SD)
*Primary outcome*												
Risk relative to individual of the same age and sex with a recommended lifestyle	253	1.5 (0.8)	−0.0001 (0.6)	252	1.6 (1.1)	−0.07 (0.9)	256	1.5 (0.8)	0.007 (0.4)	257	1.5 (0.8)	0.003 (0.4)

*Secondary continuous outcomes*												
Awareness of cancer risk factors	238	40.0 (5.6)	−1.6 (4.5)	241	40.9 (5.0)	−2.2 (4.3)	237	40.3 (5.7)	−1.9 (4.6)	242	40.7 (5.6)	−2.2 (4.5)
Risk conviction												
Absolute	247	2.9 (1.5)	0.06 (1.8)	250	3.0 (1.7)	−0.17 (1.5)	253	2.9 (1.6)	−0.47 (1.7)	256	2.9 (1.5)	0.10 (1.5)
Comparative	243	3.2 (1.5)	0.19 (1.5)	245	3.3 (1.6)	−0.09 (1.6)	249	3.2 (1.5)	−0.14 (1.4)	245	3.2 (1.5)	0.03 (1.6)
Maladaptive coping	250	9.2 (2.1)	0.25 (1.9)	249	9.3 (2.0)	0.51 (1.9)	255	9.4 (2.0)	0.32 (1.8)	256	9.2 (2.1)	0.16 (1.9)
Anxiety (SF-SSAI)	242	12.0 (4.2)	−0.60 (3.2)	245	12.2 (4.4)	−0.75 (3.7)	252	12.3 (4.3)	−0.38 (3.5)	246	12.0 (3.9)	−0.42 (3.1)
Cancer worry	248	5.0 (2.2)	−0.06 (2.0)	249	5.4 (2.6)	−0.02 (2.0)	252	5.3 (2.6)	−0.24 (2.1)	252	5.3 (2.6)	−0.01 (1.9)
Lifestyle												
BMI	253	28.5 (6.7)	−0.42 (3.9)	252	28.7 (7.0)	−0.17 (3.8)	256	28.2 (7.1)	0.04 (3.7)	257	28.0 (6.8)	−0.19 (3.4)
Alcohol	251	5.9 (9.4)	0.35 (4.6)	250	5.7 (9.0)	−0.005 (4.8)	254	5.3 (7.8)	−0.22 (5.0)	256	5.5 (8.8)	0.23 (3.7)
Physical activity	243	3.4 (3.1)	0.36 (2.4)	241	3.1 (2.8)	0.66 (2.5)	243	3.2 (3.0)	0.48 (2.4)	245	3.5 (3.2)	0.54 (2.8)
Fruit and veg	253	2.0 (1.5)	0.23 (1.4)	252	1.9 (1.3)	0.30 (1.4)	256	1.9 (1.4)	0.31 (0.94)	257	1.9 (1.4)	0.27 (0.93)
Red meat	253	2.1 (1.7)	0.10 (1.5)	251	2.1 (2.0)	−0.10 (1.2)	255	1.9 (1.6)	0.15 (1.6)	257	2.0 (1.8)	−0.02 (1.5)
Processed meat	253	2.0 (1.8)	−0.06 (1.2)	252	2.0 (2.1)	−0.12 (1.4)	256	1.8 (1.7)	0.05 (1.6)	257	1.8 (1.6)	−0.08 (1.3)


	Group 1: Lifestyle advice			Group 2: Bar charts			Group 3: Pictographs			Group 4: Qualitative		
	Baseline		Change^a^ from baseline	Baseline		Change^a^ from baseline	Baseline		Change^a^ from baseline	Baseline		Change^a^ from baseline
	n	% (n)	%	n	% (n)	%	n	% (n)	%	n	% (n)	%

*Secondary categorical outcomes*												
Current smoker	253	15 (39)	0.5	252	12 (31)	2.8	256	15 (38)	0	257	14 (37)	0.5
Risk accuracy												
Absolute	223	19 (42)	−6.4	227	21 (47)	−10.5	225	19 (42)	−1.8	236	18 (42)	−4.5
Comparative	253	37 (94)	6.3	252	40 (100)	−3.3	256	43 (110)	0.5	257	41 (106)	−10.4

a Change from baseline was calculated as three month follow-up minus baseline.

There was no evidence of differences in change from baseline RRI between the randomised groups at three-month follow up (*p* = 0.711). There was a significant interaction with baseline RRI (*p* < 0.001) but no interactions between the interventions and age (*p* = 0.183), sex (*p* = 0.486), self-perceived risk at baseline below or above computed risk (*p* = 0.713) or numeracy (*p* = 0.886).

### 3.3 Secondary outcomes

At immediate follow-up there were significant increases in absolute and comparative risk conviction, response efficacy, intention to consume more fruit and vegetables, intention to reduce processed meat consumption and absolute risk accuracy in the three intervention groups combined compared with the lifestyle only group (Fig. 4 and Table 2). Consistent with the fact that those in the qualitative group did not receive numbers relating to their absolute risk, there were also increases in absolute risk conviction and absolute risk accuracy in the bar chart group compared with the qualitative group, absolute risk conviction in the pictograph group compared with the qualitative group and absolute risk accuracy in the bar chart group compared with the qualitative group.

For both absolute and comparative risk conviction there was a significant interaction with baseline risk conviction (*p* = 0.002 and *p* = 0.007 respectively), such that the increases in risk conviction emerged only amongst those with low baseline conviction. There was also a significant interaction with baseline risk conviction for comparative risk accuracy (*p* = 0.003), such that increases in comparative risk accuracy observed in the intervention groups relative to the control and the pictograph group relative to the bar chart group appeared only in those with high baseline risk conviction. For absolute risk accuracy there were interactions with both numeracy (*p* = 0.009) and whether the participants were accurate at baseline (*p* = 0.001). No differences were seen in those with low numeracy or who were accurate at baseline. For those with high numeracy or who overestimated their risk at baseline all four comparisons were significant. There were no interactions with age or sex.

At 3-month follow-up there was a significant difference in comparative risk accuracy between the four groups, with an increase in accuracy in the pictograph group compared with the qualitative scale group. As at immediate follow-up, there was a significant interaction with baseline risk conviction (*p* = 0.002), with the increase only observed in those with low baseline risk conviction. There was also a significant interaction for comparative risk accuracy with numeracy (*p* = 0.036) and for absolute risk accuracy with baseline risk conviction (*p* = 0.037), but no between group differences. There were no other between-group differences in any of the measured outcomes (Fig. 5 and Table 3). No differences were seen in sensitivity analyses using different thresholds to define accuracy.

### 3.4 Acceptability and process measures

The acceptability of the cancer-specific lifestyle advice was good with the majority of participants finding it understandable (84%), trustworthy (75%), useful (77%), motivating (67%), important (74%) and well presented (78%) (Supplementary Table S4). The acceptability of the interventions was similarly high.

Of the six cancer-specific lifestyle advice pages with which participants were provided, 60% did not view any and only 21% viewed three or more (Supplementary Table S5). There was no significant difference (*p* = 0.379) in the number of lifestyle pages viewed across the four groups and no significant differences in intention or behaviour between the groups in the post hoc analysis including only the participants who had viewed one or more lifestyle advice pages (Supplementary Tables S6 and S7). The control group spent on average 46 seconds (s) (IQR 30 s–1m 35 s) viewing the lifestyle information pages. This compares with an average 2 minutes (min) 24 seconds (s) (IQR 1 m 34 s–3 m 47 s) the intervention groups spent interacting with the intervention and viewing the same risk pages (Supplementary Table S5).

## 4 Discussion

Our findings suggest that the provision of personalised cancer risk information alongside lifestyle advice can increase short term risk accuracy, conviction about one's risk, and intention to change behaviour more than lifestyle advice alone. It appears to do so without increasing cancer related worry or anxiety yet has little impact on health-related behaviour. Our results also suggest that the impact of risk communication on risk perception may be dependent on format and recipients' numeracy and the conviction with which they believe their initial perceived risk.

Similar increases in short term risk accuracy have been demonstrated previously (Bayne et al., 2020). Of the 13 studies identified in that review, however, only one reported risk accuracy at two time points (Rimer et al., 2002) and only two compared provision of absolute and comparative risk estimates (Timmermans and Oudhoff, 2012; Weinstein et al., 2004b). Our findings that the increases in absolute risk accuracy immediately after the intervention were only amongst those with high numeracy and those who overestimated their risk at baseline, and did not persist at three months highlight the challenges associated with changing individuals' perceptions of their own risk and the potential for risk information to widen inequalities. To our knowledge, this is not only the first study to measure change in multiple behaviours following provision of personalised cancer risk information but is also the first study to explore the effect of risk communication on risk conviction and the association between risk conviction and risk accuracy. Our findings suggest that risk conviction, as measured in this study, is distinct from risk perception. Moreover, accuracy of perceived risk after risk information appears to be moderated by risk conviction. Importantly, we also showed that risk conviction increased within the intervention groups, demonstrating that risk conviction can be manipulated. Those differences were only observed in those with low baseline risk conviction, however, suggesting that changing risk conviction is more difficult in those who already have strong beliefs about their risk. These differences were not present at three months, suggesting that changes in risk conviction may not persist.

The observed increase in intention to consume more fruit and vegetables and reduce processed meat consumption amongst participants in the intervention groups suggests that provision of risk information may have short-term beneficial effects over and above lifestyle information alone. These differences were, however, small and were not associated with health-behaviour change. This lack of behaviour change may reflect the low levels of engagement with the lifestyle advice components but is also consistent with previous studies (French et al., 2017) and the well-reported gap between intention and behaviour change (Usher-Smith et al., 2018a; Walker et al., 2015).

It is also possible that participants did not understand the risk estimates or the meaning of cancer risk: a substantial proportion reported perceived absolute cancer risk of 50% both before and after the intervention, potentially reflecting uncertainty (Bruine de Bruin and Carman, 2012).

Additionally, as we hypothesised and is seen in previous studies (Bayne et al., 2020; Weinstein et al., 2004b), the majority (81%) of participants over-estimated their absolute cancer risk at baseline. As well as potentially reducing maladaptive behaviours, conceivably the risk information could have reassured participants, leading to the adoption of unhealthy behaviours. Our results show no evidence of this. They additionally support existing evidence that provision of disease risk does not increase disease-specific worry or general anxiety (Silarova et al., 2019; Li et al., 2016; Godino et al., 2012).

### 4.1 Strengths and limitations

Our study benefits from several key strengths. These include the randomised control trial design, the large sample size and the high retention at three-month follow-up.

However, we acknowledge some weaknesses. In particular, using an online platform enabled recruitment of a large sample size but limited external validity: the demographics of Prolific members are not necessarily representative of the UK population (Palan and Schitter, 2018). We were, however, able to recruit a population with a range of ages and educational attainment. The median computed risk relative to an average individual of the same age and sex was 1 (IQR 0.8–1.2), indicating that most participants had a computed 10-year cancer risk comparable to that of an average individual of their age and sex in England. The observed changes in risk accuracy are also consistent with our prior hypotheses, suggesting that participants in the intervention groups engaged with the risk information. Nevertheless, the same findings may not emerge if cancer risk information were provided as part of routine clinical practice. Most of our population were also concerned about developing cancer at baseline. The response to risk information may vary depending on that a priori awareness of the health threat in other populations. The use of self-report measures of behaviour also introduces the risk of both social desirability and recall bias. Less than half of the participants engaged with the lifestyle advice component and we do not know how the presentation of the combined risk of one or more of the cancers was interpreted by participants. The findings relating to risk conviction are also based on two recently developed questions to measure risk conviction. Further research is needed to assess the validity of those questions.

## 5 Conclusion

This trial shows that provision of personalised cancer risk information alongside lifestyle advice increases risk accuracy, risk conviction and intention to change behaviour immediately without increasing anxiety or worry but there is no evidence that the information motivates health behaviour change. It additionally provides some indication of the more effective formats of risk presentation but suggests that their impact is dependent on numeracy and baseline risk conviction.

The following are the supplementary data related to this article.

Supplementary file 1 Information about risk scores provided to participants. Supplementary file 1

Supplementary file 2 Participant information sheet. Supplementary file 2

Supplementary tables Image 1

## Ethical approval

This study obtained ethical approval from the Psychology Research Ethics committee of the University of Cambridge on 12 December 2017 (Ref: PRE.2017.093).

## Availability of data and materials

All the data will be stored in accordance with the Data Protection Act 1998 within the University of Cambridge data repository (https://www.repository.cam.ac.uk/) for at least 10 years from the last access. Two years after completion of the trial all anonymised data will be shared via the repository.

## Funding

This study was funded by a 10.13039/501100000289Cancer Research UK Prevention Fellowship (C55650/A21464).

GM is supported by an NIHR Academic Clinical Fellowship.

SJS is supported by the 10.13039/501100000265Medical Research Council (unit programme no MC_UU_12015/1). The University of Cambridge has received salary support in respect of SJG from the NHS in the East of England through the Clinical Academic Reserve. The views expressed in this publication are those of the authors and not necessarily those of the NHS, the NIHR or the Department of Health or the U.S. National Institutes of Health.

All researchers were independent of the funding bodies and the funders had no role in data collection, analysis and interpretation of data; in the writing of the report; or decision to submit the article for publication.

## CRediT authorship contribution statement

**Golnessa Masson:** Conceptualization, Methodology, Software, Formal analysis, Resources, Data curation, Writing - original draft, Visualization. **Katie Mills:** Methodology, Resources, Writing - review & editing, Project administration. **Simon J. Griffin:** Conceptualization, Methodology, Writing - review & editing, Supervision, Funding acquisition. **Stephen J. Sharp:** Methodology, Software, Formal analysis, Writing - review & editing. **William M.P. Klein:** Conceptualization, Methodology, Writing - review & editing. **Stephen Sutton:** Conceptualization, Methodology, Writing - review & editing, Funding acquisition. **Juliet A. Usher-Smith:** Conceptualization, Methodology, Software, Validation, Formal analysis, Investigation, Resources, Data curation, Writing - original draft, Visualization, Supervision, Funding acquisition.

## Declaration of competing interest

The authors declare that there are no conflicts of interest.
