# Supplementary material for: A randomised controlled trial of the effect of providing online risk information and lifestyle advice for the most common preventable cancers
Source: Prev Med. 2020 Sep;138:106154. doi: 10.1016/j.ypmed.2020.106154 (PMC7378571; doi:10.1016/j.ypmed.2020.106154)
Supplement: Supplementary file 1 — Information about risk scores provided to participants. [file mmc1.pdf]

About the cancer risk estimates

In the UK, **1 in 2** people will develop cancer in their lifetime. Although half of people with cancer live 10 years after diagnosis, cancer still causes more than **1 in 4** deaths. Treatment can also be unpleasant. Preventing cancer is therefore important.

Many people feel whether they get cancers is purely down to their genes or bad luck but experts now know that **more than 4 in 10** cancers could be prevented if people led healthier lifestyles.

Today you have been given the cancer risk of a person of the same age, sex, bodyweight and lifestyle factors as you.

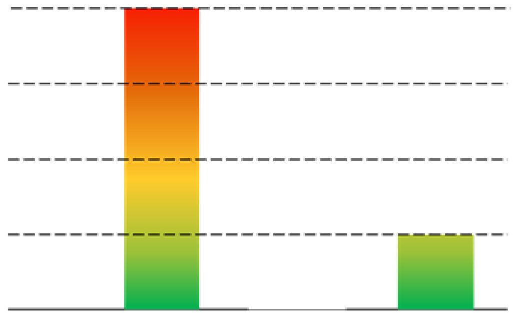

Where do the estimates of cancer risk come from?

The estimates are calculated based on data from lots of studies which have looked at how lifestyle affects risk of cancer.

How accurate are the estimates?

We have tested the estimates within a population of over 20,000 people living in the East of England and they apply well to that group. However, they do not apply perfectly to all individuals. A high risk estimate does not mean that you are destined to get cancer and a low risk estimate does not mean that you are not going to get cancer. There are also other factors, including other diseases or medications that you take, that can affect the risk of developing cancer.

Why did you choose these five cancers?

There are more than 200 different types of cancer. In order to show the effect of lifestyle we have included the five most common ones that are affected by lifestyle. Adopting a healthy lifestyle will also reduce the risk of developing other types of cancer.

Who can I talk to about my cancer risk?

If you would like to talk to someone about your future risk of cancer please contact your GP to make an appointment.
