# Supplementary material for: A randomised controlled trial of the effect of providing online risk information and lifestyle advice for the most common preventable cancers
Source: Prev Med. 2020 Sep;138:106154. doi: 10.1016/j.ypmed.2020.106154 (PMC7378571; doi:10.1016/j.ypmed.2020.106154)
Supplement: Supplementary file 2 — Participant information sheet. [file mmc2.pdf]

I-CaPP

Interventions for Cancer Prevention in Primary Care

Online risk presentation study
Participant Information Sheet

Thank you for considering taking part in our online research study to help the development of personalised cancer risk tools. Please take time to read this information carefully to understand why the research is being done and what it will involve for you.

Who is organising the research?
The study is being led by doctors and researchers at the University of Cambridge. It is funded by Cancer Research UK and has been reviewed and given a favourable ethical opinion by the Psychology Research Ethics Committee of the University of Cambridge.

What is the purpose of the study?
In this study, we are hoping to learn more about how people respond to receiving information about their risk of developing cancer and ways to reduce that risk.

Who is taking part?
We are inviting UK residents aged 30-74 years who are registered with Prolific.

Do I have to take part?
No, you can withdraw from this study at any time without explanation and there will be no consequences for doing so.

What happens next?
You will first be asked to complete an online consent form. Then you will answer a set of questions about your diet, lifestyle and beliefs about your risk of cancer. You will then either be presented with your personalised 10-year cancer risk and how this might be affected by lifestyle change before seeing information about lifestyle change or will go straight to the information about lifestyle change without seeing your personalised risk. This will allow us to compare the responses from people who see their personalised cancer risk with those who don't. Which of these groups you are in will be determined at random by a computer program. You will not be able to choose which of these two groups you are allocated to. You will then answer a further set of questions about your thoughts about your risk of cancer and motivation to make lifestyle change. Lastly, you will be asked if you agree to take part in a follow up task 3 months later where you will answer a similar set of questions to check how your lifestyle and ideas about cancer risk have changed with time. After completing that task, if you were one of the people who did not see their personalised risk in the first task, you will be offered the opportunity to see it then.

How much time will it take?
The first part of the task will take approximately 20 minutes to complete. The 3-month follow up part is shorter and will take approximately 10 minutes to complete.

What are the possible benefits of taking part?

By taking part in this study, you will receive a summary of your estimated risk of developing one of the five most common preventable cancers in the future and information about how you may be able to reduce that risk. It is also an opportunity to contribute to and help influence cancer risk research by sharing your experiences.

Are there any disadvantages or risks of taking part?

Being presented with your risk of developing cancer can be a sensitive or stressful issue. If you become distressed by anything when completing the task you may exit it at any stage and there are no consequences associated with doing so. If you have concerns about your risk of developing cancer you can also contact your GP or find further information on cancer and support organisations on the Cancer Research UK or Macmillan Cancer Support websites (www.cancerresearchuk.org; www.macmillan.org.uk).

Will I be paid for taking part?

Yes, there is a £3 (£6 per hour) reward for the time you spend completing this task. £2 will be paid to you through Prolific once your response to the first part has been accepted by the researchers and an additional £1 will be paid to you once you have completed the follow-up part in 3 months.

Will my taking part in this study be kept confidential?

All information that is collected from you during the course of the study is anonymised and stored securely. Prolific will not share any personal or identifiable information about you with the researchers.

What will happen to the results of the study?

The results of the study will be presented at scientific meetings and in scientific journals. You will not be able to be identified in these publications. After the study we will send you a summary of the results via Prolific. Anonymous information collected as part of the research may also be shared with responsible individuals from the sponsor, regulatory authorities and researchers.

What if there is a problem or something goes wrong?

If you have any concerns or complaints about any aspect of this study you can telephone the study lead, Dr Juliet Usher-Smith, on 01223 748693 or email her at: jau20@medschl.cam.ac.uk

Alternatively, if you would like to write to her, please send your letter to:

Dr Juliet Usher-Smith
The Cancer Risk Study
University of Cambridge
Department of Public Health and Primary Care
Box 113 Cambridge Biomedical Campus
Cambridge CB2 0SR

We will reply to your email or letter promptly in writing, unless you enclose your telephone number and wish to discuss your concerns by telephone.

Thank you very much for reading this information leaflet and choosing to take part in this study
