# Supplementary material for: A randomised controlled trial of the effect of providing online risk information and lifestyle advice for the most common preventable cancers
Source: Prev Med. 2020 Sep;138:106154. doi: 10.1016/j.ypmed.2020.106154 (PMC7378571; doi:10.1016/j.ypmed.2020.106154)
Supplement: Supplementary file 3 — Supplementary tables [file mmc3.docx]

**Supplementary Table S1. Outcome measures at each time point in the trial**

| Measure | Details | Pre | Post | 3 months post |
| --- | --- | --- | --- | --- |
| Demographics |  |  |  |  |
| Age | Years | ✓ | --- | ✓ |
| Sex | Male/female | ✓ | --- | ✓ |
| Ethnicity | 1 = White  2 = Mixed/ Multiple ethnic group  3 = Asian/ Asian British  4 = Black/ African/ Caribbean  5 = Other | ✓ | --- | --- |
| Family history of cancer | Assessed by one question “Have your parents or any brothers or sisters ever had cancer?” | ✓ | --- | --- |
| Highest education level | 1 = No formal education  2 = Primary education  3 = Secondary education  4 = University education | ✓ | --- | --- |
| Lifestyle |  |  |  |  |
| Weight |  | ✓ | --- | ✓ |
| Height |  | ✓ | --- | --- |
| Smoking status | Non smoker / Ex-smoker / Current smoker | ✓ | --- | ✓ |
| Alcohol | Units per week | ✓ | --- | ✓ |
| Physical activity | Hours per week | ✓ | --- | ✓ |
| Fruit | Portions per day | ✓ | --- | ✓ |
| Vegetables | Portions per day | ✓ | --- | ✓ |
| Red meat | Portions per week | ✓ | --- | ✓ |
| Processed meat | Portions per week | ✓ | --- | ✓ |
| Primary outcome |  |  |  |  |
| Risk relative to | Change from baseline to three months in computed 10-year risk of developing one of the five commonest preventable cancers ((lung, colorectal, bladder, kidney and oesophageal for men and breast, lung, colorectal, endometrial and kidney for women) relative to an individual of the same age and sex with a recommended lifestyle. The computed risk was calculated using a lifestyle-based risk score incorporating body mass index, smoking status, alcohol consumption, physical activity, fruit and vegetable consumption and red and processed meat consumption. Full details of the development and validation of the lifestyle risk score have been published elsewhere(1) | ✓ | --- | ✓ |
| Secondary outcomes |  |  |  |  |
| Awareness of cancer risk factors | Questions 6 and 8 from the CR-UK Perceptions of risk survey(2) | ✓ | ✓ | ✓ |
| Risk perception | Absolute and comparative perception of risk using both a continuous and categorical measure as in previous studies(3) adapted for cancer (e.g. “On a scale from 0 to 100%, how would you rate the probability that you will develop one of these five cancers in the next 10 years?”, and “How do you think your chance of developing one of these five cancers in the next 10 years compares to the average person of your sex and age?”) | ✓ | ✓ | ✓ |
| Risk conviction | Two questions on a likert scale from 1 “Not at all certain/confident” to 7 “Extremely certain/confident”: “How certain are you about your answer to the above question?” and “How confident are you that the estimate you have given is accurate, that is, that it reflects your actual risk?” | ✓ | ✓ | ✓ |
| Self-efficacy | Assessed using 3 Likert items for each of physical activity and fruit and vegetables evaluated on a 5-point response scale ranging from “strongly disagree” to “strongly agree” as used in previous research(4) adapted for use in the context of cancer (e.g. “I would like to be physically active but I don’t know if I can.”, “I feel confident in my ability to be active at a moderate intensity for at least 30 minutes per day on at least 5 days a week.”, and “I am confident that I could be physically active if I wanted to.”. | --- | ✓ | --- |
| Response-efficacy | Assessed using 3 Likert items for each of physical activity and fruit and vegetables evaluated on a 5-point response scale ranging from “strongly disagree” to “strongly agree” as used in previous research(4) adapted for use in the context of cancer (e.g. ‘Eating a healthy diet is effective in preventing cancer”, “Eating a healthy diet works in preventing cancer”, and “If I eat a healthy diet, I am less likely to develop cancer”. | --- | ✓ | --- |
| Maladaptive coping | Assessed using three questions measuring avoidance, fatalism and hopelessness as used in previous research(5): “I try not to think about the possibility of developing cancer.” [*Avoidance*] “If you are destined to develop cancer you will, there is really very little you can do about it.” [*Fatalism*] “Given what I know about cancer, I sometimes feel it is almost useless to try to stay healthy.” [*Hopelessness*] | ✓ | ✓ | ✓ |
| Intention to change behaviour | Overall intention assessed using four questions: “I am determined to do everything I can to avoid getting cancer in the future.” “I am committed to engaging in behaviours that protect me against getting cancer in the future.” “I fully intend to have a lifestyle that will prevent me from getting cancer in the future.” “I will try to do all I can to avoid getting cancer in the future.” evaluated on a 7-point response scale from “Strongly agree” to “Strongly disagree” and treated as a continuous variable. Specific intention assessed using questions for each behaviour e.g. “I intend to be more physically active in the next 3 months”) evaluated on a 7-point response scale from “Strongly agree” to “Strongly disagree”, again each treated as continuous variables. | --- | ✓ | --- |
| Worry | The Lerman Cancer Worry Scale(6) | ✓ | --- | ✓ |
| Anxiety | Short form of the Spielberger State Trait Anxiety Inventory (STAI)(7) | ✓ | ✓ | ✓ |
| Cognitive evaluation of provision of cancer risk scores: | |  |  |  |
| Acceptability | How much they agree that the intervention was useful, understandable, trustworthy, motivating, and important in helping them decide about cancer risk reduction as used previously(8) | --- | ✓ | --- |
| Potential mediators and moderators | |  |  |  |
| Numeracy | The 3-item Schwartz scale(9) | ✓ | --- | --- |
| Time orientation | The brief nine item form of the Zimbardo Time Perspective Inventory (ZTPI-R)(10) | ✓ | --- | --- |
| Self-rated general health | Assessed with one question “How would you rate your general health?” on a 5-point scale from “Very good” to “Poor” | ✓ | --- | --- |
| Previous information on cancer | Assessed with one question “Have you ever received information on your risk of developing cancer?” | ✓ | --- | --- |
| Concern about developing cancer | Assessed with one question “How concerned are you about developing cancer in your lifetime?” on a likert scale from 1 “Not at all” to 7 “Extremely” | ✓ | --- | --- |

1. Usher-Smith JA, Sharp SJ, Luben R, Griffin SJ. Development and validation of lifestyle-based models to predict incidence of the most common potentially preventable cancers. Cancer Epidemiol Biomarkers Prev. 2018;cebp.0400.2018.

2. Cancer Research UK. Perceptions of Risk Survey 2008 : Key Findings. 2008; Available from: www.cancerresearchuk.org/sites/default/files/perceptions_of_risk_survey.pdf

3. Godino JG, van Sluijs EMF, Sutton S, Griffin SJ. Understanding perceived risk of type 2 diabetes in healthy middle-aged adults: a cross-sectional study of associations with modelled risk, clinical risk factors, and psychological factors. Diabetes Res Clin Pract. 2014;106(3):412–9.

4. Sanderson SC, Persky S, Michie S. Psychological and Behavioral Responses to Genetic Test Results Indicating Increased Risk of Obesity: Does the Causal Pathway from Gene to Obesity Matter? Public Health Genomics. 2010;13(1):34–47.

5. Rippetoe P a, Rogers RW. Effects of components of protection-motivation theory on adaptive and maladaptive coping with a health threat. J Pers Soc Psychol. 1987;52(3):596–604.

6. Lerman C, Trock B, Rimer BK, Jepson C, Brody D, Boyce A. Psychological side effects of breast cancer screening. Heal Psychol. 1991;10(4):259–67.

7. Marteau TM, Bekker H. The development of a six-item short-form of the state scale of the Spielberger State-Trait Anxiety Inventory (STAI). Br J Clin Psychol. 1992 Sep;31 ( Pt 3):301–6.

8. Sheridan SL, Draeger LB, Pignone MP, Sloane PD, Samuel-Hodge C, Finkelstein E a., et al. Designing and implementing a comparative effectiveness study of two strategies for delivering high quality CHD prevention: Methods and participant characteristics for the Heart to Health study. Contemp Clin Trials. 2013;36(2):394–405.

9. Schwartz LM, Woloshin S, Black WC, Welch HG. The role of numeracy in understanding the benefit of screening mammography. Ann Intern Med. 1997;127(11):966–72.

10. Crockett RA, Weinman J, Hankins M, Marteau T. Time orientation and health-related behaviour: Measurement in general population samples. Psychol Heal. 2009;24(3):333–50.

**Supplementary Table S2**. Pairwise comparisons for secondary outcomes at immediate follow-up

|  | **Control vs all intervention groups** | **Bar chart vs pictographs** | **Bar chart vs qualitative scale** | **Pictographs vs qualitative scale** |  |  |
| --- | --- | --- | --- | --- | --- | --- |
| **Secondary continuous outcomes** | **Difference**  **(98.75% CI)** | **Difference**  **(98.75% CI)** | **Difference**  **(98.75% CI)** | **Difference**  **(98.75% CI)** | ***p* value** | ***n* in model** |
| Awareness of cancer risk factors | -0.75 (-1.50 to -0.01) | -0.11 (-1.02 to 0.80) | 0.15 (-0.77 to 1.06) | 0.25 (-0.66 to 1.17) | 0.078 | 882 |
| Risk conviction |  |  |  |  |  |  |
| Absolute | **-0.34 (-0.57 to -0.11)** | -0.22 (-0.50 to 0.06) | **0.29 (0.01 to 0.57)** | **0.51 (0.23 to 0.78)** | **<0.001** | 962 |
| Low baseline absolute risk conviction | **-0.42 (-0.70 to -0.15)** | -0.28 (-0.62 to 0.05) | **0.44 (0.10 to 0.77)** | **0.72 (0.39 to 1.05)** | **<0.001** | 644 |
| High baseline absolute risk conviction | -0.18 (-0.58 to 0.22) | -0.15 (-0.64 to 0.34) | -0.11 (-0.60 to 0.39) | 0.04 (-0.45 to 0.53) | 0.589 | 318 |
| Comparative | **-0.38 (-0.60 to -0.16)** | 0.01 (-0.26 to 0.27) | 0.12 (-0.15 to 0.38) | 0.11 (-0.16 to 0.37) | **0.002** | 916 |
| Low baseline comparative risk conviction | **-0.63 (-0.93 to -0.33)** | 0.14 (-0.21 to 0.49) | 0.27 (-0.08 to 0.62) | 0.13 (-0.23 to 0.48) | **<0.001** | 526 |
| High baseline comparative risk conviction | -0.04 (-0.36 to 0.28) | -0.19 (-0.59 to 0.20) | -0.12 (-0.53 to 0.28) | 0.07 (-0.34 to 0.47) | 0.661 | 390 |
| Self-efficacy | -0.57 (-1.38 to 0.23) | -0.003 (-0.97 to 0.97) | -0.20 (-1.17 to 0.77) | -0.20 (-1.16 to 0.76) | 0.314 | 953 |
| Response efficacy | **-0.93 (-1.74 to -0.12)** | -0.10 (-1.08 to 0.88) | 0.04 (-0.94 to 1.02) | 0.14 (-0.84 to 1.13) | **0.038** | 938 |
| Maladaptive coping | 0.17 (-0.12 to 0.45) | -0.01 (-0.36 to 0.33) | -0.05 (-0.39 to 0.30) | -0.03 (-0.38 to 0.31) | 0.519 | 955 |
| Anxiety (SF-SSAI) | 0.19 (-0.21 to 0.58) | 0.01 (-0.47 to 0.48) | 0.11 (-0.36 to 0.59) | 0.11 (-0.37 to 0.58) | 0.614 | 894 |
| Intention to change behaviour |  |  |  |  |  |  |
| General | -0.09 (-0.93 to 0.74) | 0.17 (-0.85 to 1.18) | 0.33 (-0.68 to 1.34) | 0.16 (-0.85 to 1.17) | 0.864 | 968 |
| Weight | -0.05 (-0.25 to 0.14) | 0.04 (-0.20 to 0.28) | 0.08 (-0.16 to 0.32) | 0.04 (-0.21 to 0.28) | 0.754 | 860 |
| Alcohol | -0.01 (-0.24 to 0.21) | 0.14 (-0.13 to 0.41) | 0.26 (-0.02 to 0.54) | 0.12 (-0.15 to 0.40) | 0.145 | 736 |
| Physical activity | -0.11 (-2.34 to 2.13) | -0.67 (-3.39 to 2.06) | -0.29 (-3.00 to 2.43) | 0.38 (-2.33 to 3.08) | 0.942 | 986 |
| Fruit and veg | **-0.15 (-0.30 to -0.01)** | 0.11 (-0.07 to 0.29) | 0.03 (-0.15 to 0.21) | -0.08 (-0.26 to 0.10) | **0.026** | 972 |
| Red meat | -0.15 (-0.35 to 0.05) | 0.09 (-0.16 to 0.34) | 0.08 (-0.17 to 0.33) | -0.01 (-0.26 to 0.24) | 0.231 | 832 |
| Processed meat | **-0.22 (-0.42 to -0.02)** | -0.04 (-0.28 to 0.21) | 0.06 (-0.19 to 0.30) | 0.10 (-0.15 to 0.35) | **0.033** | 832 |
|  |  |  |  |  |  |  |
| **Secondary categorical outcomes** | **OR**  **(98.75% CI)** | **OR**  **(98.75% CI)** | **OR**  **(98.75% CI)** | **OR**  **(98.75% CI)** | ***p* value** | ***n* in model** |
| Intention to quit smoking | 0.75 (0.52 to 1.10) | 1.00 (0.63 to 1.59) | 1.05 (0.65 to 1.70) | 1.06 (0.67 to 1.67) | 0.299 | 266 |
| Risk accuracy |  |  |  |  |  |  |
| Absolute | **0.26 (0.12 to 0.57)** | **0.22 (0.11 to 0.44)** | **3.46 (1.41 to 8.50)** | **15.83 (6.67 to 37.36)** | **<0.001** | 817 |
| Low numeracy | 0.51 (0.12 to 2.11) | 0.26 (0.05 to 1.34) | 0.98 (0.16 to 5.79) | 3.69 (0.70 to 19.38) | 0.057 | 206 |
| High numeracy | **0.21 (0.08 to 0.55)** | **0.21 (0.10 to 0.47)** | **5.89 (1.90 to 18.28)** | **27.39 (9.16 to 81.93)** | **<0.001** | 609 |
| Underestimate at baseline | **---** | **---** | **---** | **---** | **---** | 2 |
| Accurate at baseline | 0.66 (0.21 to 2.08) | 0.85 (0.17 to 4.20) | 1.79 (0.42 to 7.66) | 2.12 (0.44 to 10.27) | 0.502 | 161 |
| Overestimate at baseline | **0.09 (0.01 to 0.60)** | **0.22 (0.11 to 0.46)** | **6.62 (1.63 to 26.85)** | **29.75 (7.88 to 112.25)** | **<0.001** | 654 |
| Comparative | 1.25 (0.81 to 1.91) | 1.58 (0.93 to 2.68) | 1.20 (0.71 to 2.02) | 0.76 (0.45 to 1.29) | 0.103 | 982 |
| Low baseline comparative risk conviction | 0.90 (0.50 to 1.59) | 1.22 (0.62 to 2.39) | 0.92 (0.47 to 1.78) | 0.75 (0.39 to 1.45) | 0.691 | 546 |
| High baseline comparative risk conviction | **2.11 (1.06 to 4.17)** | **2.67 (1.08 to 6.61)** | 2.02 (0.84 to 4.90) | 0.76 (0.31 to 1.88) | **0.003** | 436 |

**Secondary continuous outcomes:** Difference (for example, Control group minus all intervention groups) and 98.75% CI estimated from ANCOVA model with adjustment for baseline and stratification variables (age, sex and risk relative to an individual with a recommended lifestyle).

p-value from a 3 degrees of freedom (d.f.) F-test of the null hypothesis, within an ANCOVA model. Null hypothesis is that there is no difference between the four randomised groups.

**Secondary categorical outcomes:** Difference in probability of outcome and 98.75% confidence interval estimated from logistic regression model, adjusted for baseline value of the outcome and stratification variables (age, sex and risk relative to an individual with a recommended lifestyle).

p-value from a Wald test (3 d.f.) of the null hypothesis that there is no difference in probability of the outcome between the four randomised groups.

**Supplementary Table S3**. Pairwise comparisons for primary and secondary outcomes at three month follow-up

|  | **Control vs all intervention groups** | **Bar chart vs pictographs** | **Bar chart vs qualitative scale** | **Pictographs vs qualitative scale** |  |  |
| --- | --- | --- | --- | --- | --- | --- |
|  | Difference  (98.75% CI) | Difference  (98.75% CI) | Difference  (98.75% CI) | Difference  (98.75% CI) | *p* value | *n* in model |
| **Primary outcome** |  |  |  |  |  |  |
| Risk relative to individual of the same age and  sex with a recommended lifestyle (RRI) | 0.02 (-0.09 to 0.13) | -0.05 (-0.18 to 0.08) | -0.04 (-0.17 to 0.09) | 0.01 (-0.12 to 0.14) | 0.711 | 863 |
| Baseline RRI ≤ 1.5 | 0.03 (-0.04 to 0.11) | 0.02 (-0.07 to 0.10) | 0.02 (-0.07 to 0.11) | 0.004 (-0.08 to 0.09) | 0.609 | 581 |
| Baseline RRI >1.5 | -0.03 (-0.23 to 0.25) | -0.19 (-0.54 – 0.16) | -0.13 (-0.47 to 0.21) | 0.07 (-0.28 to 0.41) | 0.556 | 282 |
|  |  |  |  |  |  |  |
| **Secondary continuous outcomes** |  |  |  |  |  |  |
| Awareness of cancer risk factors | -0.23 (-1.09 to 0.63) | -0.07 (-1.11 to 0.96) | 0.02 (-1.01 to 1.05) | 0.09 (-0.94 to 1.12) | 0.920 | 755 |
| Risk conviction |  |  |  |  |  |  |
| Absolute | -0.09 (-0.37 to 0.19) | 0.13 (-0.21 to 0.46) | 0.01 (-0.32 to 0.34) | -0.12 (-0.45 to 0.22) | 0.603 | 833 |
| Comparative | -0.14 (-0.41 to 0.13) | 0.03 (-0.30 to 0.36) | 0.02 (-0.30 to 0.35) | -0.004 (-0.33 to 0.33) | 0.648 | 789 |
| Low baseline comparative risk conviction | -0.02 (-0.40 to 0.35) | 0.24 (-0.21 to 0.68) | 0.14 (-0.30 to 0.57) | -0.10 (-0.54 to 0.34) | 0.606 | 455 |
| High baseline comparative risk conviction | -0.27 (-0.68 to 0.13) | -0.28 (-0.77 to 0.22) | -0.16 (-0.66 to 0.35) | 0.12 (-0.38 t 0.62) | 0.187 | 334 |
| Maladaptive coping | 0.18 (-0.18 to 0.54) | 0.18 (-0.25 to 0.61) | 0.18 (-0.24 to 0.61) | 0.01 (-0.42 to 0.43) | 0.381 | 837 |
| Anxiety (SF-SSAI) | 0.02 (-0.62 to 0.65) | -0.14 (-0.89 to 0.61) | -0.01(-0.76 to 0.74) | 0.13 (-0.62 to 0.88) | 0.965 | 793 |
| Cancer worry | -0.07 (-0.44 to 0.30) | 0.29 (-0.15 to 0.74) | 0.06 (-0.38 to 0.50) | -0.24 (-0.67 to 0.20) | 0.341 | 831 |
| Lifestyle |  |  |  |  |  |  |
| BMI | -0.31 (-1.02 to 0.40) | -0.12 (-0.98 to 0.74) | 0.13 (-0.72 to 0.99) | 0.25 (-0.60 to 1.10) | 0.631 | 863 |
| Alcohol | 0.54 (-0.33 to 1.40) | 0.24 (-0.79 to 1.28) | -0.18 (-1.21 to 0.86) | -0.42 (-1.45 to 0.61) | 0.325 | 850 |
| Physical activity | -0.21 (-0.72 to 0.29) | 0.14 (-0.46 to 0.75) | 0.06 (-0.55 to 0.66) | -0.09 (-0.69 to 0.52) | 0.686 | 801 |
| Fruit | -0.02 (-0.25 to 0.21) | -0.01 (-0.29 to 0.26) | -0.04 (-0.24 to 0.31) | 0.05 (-0.22 to 0.33) | 0.958 | 861 |
| Vegetables | -0.03 (-0.25 to 0.19) | -0.11 (-0.38 to 0.15) | -0.06 (-0.33 to 0.20) | 0.05 (-0.22 to 0.31) | 0.751 | 862 |
| Red meat | 0.97 (-0.18 to 0.37) | -0.22 (-0.55 to 0.11) | -0.06 (-0.39 to 0.27) | 0.16 (-0.17 to 0.49) | 0.284 | 862 |
| Processed meat | -0.01 (-0.26 to 0.24) | -0.08 (-0.39 to 0.22) | 0.01 (-0.29 to 0.31) | 0.09 (-0.21 to 0.39) | 0.859 | 863 |
|  |  |  |  |  |  |  |
| **Secondary categorical outcomes** | **OR**  **(98.75% CI)** | **OR**  **(98.75% CI)** | **OR**  **(98.75% CI)** | **OR**  **(98.75% CI)** | ***p* value** | ***n* in model** |
| Quit smoking | 0.97 (0.34 to 2.74) | 2.01 (0.58 to 7.34) | 1.77 (0.51 to 6.10) | 0.86 (0.23 to 3.14) | 0.507 | 863 |
| Risk accuracy |  |  |  |  |  |  |
| Absolute | 0.82 (0.39 to 1.71) | 0.50 (0.21 to 1.19) | 0.75 (0.31 to 1.86) | 1.50 (0.65 to 3.45) | 0.195 | 691 |
| Low baseline absolute risk conviction | 0.89 (0.33 to 2.41) | 0.39 (0.11 to 1.37) | 0.60 (0.16 to 2.23) | 1.55 (0.49 to 4.87) | 0.286 | 453 |
| High baseline absolute risk conviction | 0.92 (0.29 to 2.89) | 0.62 (0.18 to 2.17) | 0.87 (0.23 to 3.33) | 1.41 (0.40 to 5.01) | 0.792 | 238 |
| Comparative | 1.42 (0.92 to 2.18) | 0.70 (0.41 to 1.18) | 1.33 (0.77 to 2.29) | **1.91 (1.12 to 3.24)** | **0.005** | 858 |
| Low baseline comparative risk conviction | 1.15 (0.65 to 2.05) | 0.48 (0.24 to 0.96) | 0.98 (0.49 to 1.96) | 2.06 (1.04 to 4.08) | 0.021 | 479 |
| High baseline comparative risk conviction | 1.96 (0.99 to 3.85) | 1.20 (0.52 to 2.76) | 2.10 (0.85 to 5.16) | 1.76 (0.72 to 4.25) | 0.019 | 379 |
| Low numeracy | 0.97 (0.43 to 2.18) | 0.92 (0.33 to 2.57) | 1.99 (0.71 to 5.61) | 2.16 (0.77 to 6.07) | 0.244 | 220 |
| High numeracy | 1.58 (0.94 to 2.64) | 0.62 (0.33 to 1.16) | 1.14 (0.60 to 2.18) | 1.84 (0.98 to 3.47) | 0.011 | 635 |

**Primary and Secondary continuous outcomes:** Difference (for example, Control group minus all intervention groups) and 98.75% CI estimated from ANCOVA model with adjustment for baseline and stratification variables (age, sex and risk relative to an individual with a recommended lifestyle).

p-value from a 3 degrees of freedom (d.f.) F-test of the null hypothesis, within an ANCOVA model. Null hypothesis is that there is no difference between the four randomised groups.

**Secondary categorical outcomes:** Difference in odds of outcome and 98.75% confidence interval estimated from logistic regression model, adjusted for baseline value of the outcome and stratification variables (age, sex and risk relative to an individual with a recommended lifestyle).

p-value from a Wald test (3 d.f.) of the null hypothesis that there is no difference in probability of the outcome between the four randomised groups.

**Supplementary Table S4.** Acceptability measures

|  | **Group 1: Lifestyle advice only (N =253)** | | | **Group 2: Bar charts (N = 252)** | | | **Group 3: Pictographs (N = 255)** | | | **Group 4: Qualitative (N = 257)** | | |
| --- | --- | --- | --- | --- | --- | --- | --- | --- | --- | --- | --- | --- |
|  | Disagree  *n* (%) | Neither  *n* (%) | Agree  *n* (%) | Disagree  *n* (%) | Neither  *n* (%) | Agree  *n* (%) | Disagree  *n* (%) | Neither  *n* (%) | Agree  *n* (%) | Disagree  *n* (%) | Neither  *n* (%) | Agree  *n* (%) |
| **Lifestyle Advice** |  |  |  |  |  |  |  |  |  |  |  |  |
| 1 Understandable | 6(2.4) | 27(11.0) | 213(86.6) | 8(3.2) | 44(17.8) | 195(79.0) | 6(2.4) | 28(11.1) | 219(86.6) | 6(2.4) | 34(13.6) | 211(84.1) |
| 2 Trustworthy | 6(2.5) | 40(16.3) | 199(81.2) | 11(4.5) | 72(29.3) | 163(66.3) | 10(4.0) | 56(22.2) | 186(73.8) | 4(1.6) | 48(19.1) | 199(79.3) |
| 3 Useful | 12(5.0) | 39(16.1) | 191(78.9) | 12(5.0) | 52(21.5) | 178(73.6) | 8(3.2) | 42(16.9) | 199(79.9) | 9(3.6) | 47(19.0) | 192(77.4) |
| 4 Motivating | 29(12.0) | 68(28.2) | 144(59.8) | 25(10.2) | 57(23.3) | 163(66.5) | 22(9.1) | 55(22.6) | 166(68.3) | 22(8.9) | 47(19.0) | 178(72.1) |
| 5 Important | 20(8.3) | 44(18.3) | 176(73.3) | 10(4.1) | 58(23.6) | 178(72.4) | 12(4.9) | 47(19.3) | 184(75.7) | 13(5.2) | 50(20.2) | 185(74.6) |
| 6 Well presented | 15(6.3) | 45(18.9) | 178(74.8) | 10(4.1) | 55(22.5) | 180(73.5) | 7(2.9) | 36(14.8) | 200(82.3) | 10(4.1) | 33(13.5) | 202(82.5) |
| **Risk Information** |  |  |  |  |  |  |  |  |  |  |  |  |
| 1 Understandable | - | - | - | 12(5.0) | 25(10.3) | 205(84.7) | 7(2.8) | 16(6.5) | 225(90.7) | 6(2.4) | 16(6.5) | 224(91.1) |
| 2 Trustworthy | - | - | - | 10(4.1) | 59(23.9) | 178(72.1) | 8(3.2) | 52(20.6) | 193(76.3) | 8(3.2) | 52(20.6) | 192(76.2) |
| 3 Useful | - | - | - | 15(6.1) | 38(15.4) | 194(78.5) | 8(3.2) | 33(13.0) | 213(83.9) | 11(4.4) | 39(15.5) | 201(80.1) |
| 4 Motivating | - | - | - | 31(12.8) | 46(18.9) | 166(68.3) | 26(10.4) | 50(20.1) | 173(69.5) | 21(8.6) | 54(22.0) | 170(69.4) |
| 5 Important | - | - | - | 12(4.9) | 47(19.3) | 184(75.7) | 15(6.0) | 42(16.8) | 193(77.2) | 14(5.7) | 56(22.9) | 175(71.4) |
| 6 Well presented | - | - | - | 15(6.3) | 47(19.6) | 178(74.2) | 11(4.5) | 33(13.4) | 202(82.1) | 11(4.6) | 28(11.7) | 201(83.8) |

**Supplementary Table S5.** Process measures

|  | **Group 1: Lifestyle advice only (N =253)** | | | **Group 2: Bar charts (N = 252)** | | | **Group 3: Pictographs (N = 255)** | | | **Group 4: Qualitative (N = 257)** | | |
| --- | --- | --- | --- | --- | --- | --- | --- | --- | --- | --- | --- | --- |
|  | 0  *n* (%) | 1-2  *n* (%) | ≥3  *n* (%) | 0  *n* (%) | 1-2  *n* (%) | ≥3  *n* (%) | 0  *n* (%) | 1-2  *n* (%) | ≥3  *n* (%) | 0  *n* (%) | 1-2  *n* (%) | ≥3  *n* (%) |
| No of pages viewed | 174(68.8) | 25(9.9) | 54(21.3) | 141(56) | 52(20.6) | 59(23.4) | 150(58.6) | 59(23.1) | 47(18.4) | 141(56) | 55(21.4) | 58(22.6) |
| Number of additional targets set | - | - | - | 177(70.8 | 55(22.0) | 18(7.2) | 176(68.8) | 65(25.4) | 15(5.9) | 170(66.7 | 69(27.1) | 16(6.3) |
| Number of written goals | 216(85.4 | 21(8.3) | 16(6.3) | 213(84.5 | 19(7.5) | 20(7.9) | 218(85.2) | 17(6.6) | 21(8.2) | 207(80.5 | 33(12.8) | 17(6.6) |
| Total intervention time (median IQR) | 0.76 (0.51-1.59) | | | 2.28 (1.41-3.33) | | | 2.49 (1.71-4.11) | | | 2.40 (1.60-3.77) | | |

**Supplementary Table S6**. Pairwise comparisons for secondary outcomes at immediate follow-up in those who viewed at least one lifestyle advice page

|  | **Control vs all intervention groups** | **Bar chart vs pictographs** | **Bar chart vs qualitative scale** | **Pictographs vs qualitative scale** |  |  |
| --- | --- | --- | --- | --- | --- | --- |
| **Secondary continuous outcomes** | **Difference**  **(98.75% CI)** | **Difference**  **(98.75% CI)** | **Difference**  **(98.75% CI)** | **Difference**  **(98.75% CI)** | ***p* value** | ***n* in model** |
| Risk conviction |  |  |  |  |  |  |
| Absolute | **-0.43 (-0.81 to -0.05)** | -0.22 (-0.62 to 0.18) | 0.27 (-0.13 to 0.66) | **0.48 (0.08 to 0.88)** | **<0.001** | 393 |
| Low baseline absolute risk conviction | **-0.56 (-1.01 to -0.10)** | -0.11 (-0.57 to 0.36) | **0.60 (0.14 to 1.07)** | **0.71 (0.24 to 1.18)** | **<0.001** | 280 |
| High baseline absolute risk conviction | -0.10 (-0.78 to 0.57) | -0.58 (-1.34 to 0.18) | -0.73 (-1.50 to 0.04) | -0.15 (-0.91 to 0.60) | 0.090 | 113 |
| Comparative | **-0.44 (-0.80 to -0.07)** | 0.10 (-0.28 to 0.48) | 0.21 (-0.17 to 0.59) | 0.11 (-0.28 to 0.50) | **0.013** | 381 |
| Intention to change behaviour |  |  |  |  |  |  |
| General | -0.71 (-2.05 to 0.64) | -0.04 (-1.46 to 1.38) | 0.38 (-1.01 to 1.77) | 0.42 (-1.00 to 1.83) | 0.494 | 389 |
| Weight | -0.04 (-0.30 to 0.37) | -0.14 (-0.50 to 0.21) | -0.04 (-0.39 to 0.31) | 0.10 (-0.25 to 0.46) | 0.760 | 352 |
| Alcohol | -0.07 (-0.45 to 0.31) | 0.15 (-0.25 to 0.54) | 0.44 (0.03 to 0.84) | 0.29 (-0.11 to 0.69) | 0.051 | 303 |
| Physical activity | -0.72 (-2.94 to 1.50) | 0.87 (-1.45 to 3.20) | 0.16 (-2.13 to 2.44) | -0.72 (-3.03 to 1.60) | 0.642 | 398 |
| Fruit and veg | -0.10 (-0.35 to 0.14) | 0.09 (-0.16 to 0.35) | 0.04 (-0.22 to 0.29) | -0.06 (-0.31 to 0.20) | 0.570 | 395 |
| Red meat | -0.06 (-0.40 to 0.28) | 0.07 (-0.29 to 0.44) | 0.11 (-0.25 to 0.46) | 0.03 (-0.33 to 0.40) | 0.859 | 335 |
| Processed meat | -0.11 (-0.45 to 0.22) | -0.57 (-0.42 to 0.31) | 0.11 (-0.24 to 0.46) | 0.16 (-0.20 to 0.53) | 0.563 | 329 |
|  |  |  |  |  |  |  |
| **Secondary categorical outcomes** | **OR**  **(98.75% CI)** | **OR**  **(98.75% CI)** | **OR**  **(98.75% CI)** | **OR**  **(98.75% CI)** | ***p* value** | ***n* in model** |
| Intention to quit smoking | 1.00 (0.49 to 2.07) | 0.95 (0.44 to 2.06) | 1.00 (0.44 to 2.31) | 1.06 (0.48 to 2.32) | 0.998 | 91 |
| Risk accuracy |  |  |  |  |  |  |
| Absolute | **0.18 (0.04 to 0.83)** | 0.40 (0.14 to 1.17) | **4.70 (1.20 to 18.46)** | **11.75 (3.03 to 45.49)** | **<0.001** | 263 |
| Comparative | 1.00 (0.45 to 2.19) | 1.46 (0.65 to 3.26) | 1.61 (0.72 to 3.56) | 1.10 (0.49 to 2.48) | 0.481 | 398 |
| Low baseline comparative risk conviction | 0.91 (0.34 to 2.46) | 0.75 (0.27 to 2.11) | 0.92 (0.34 to 2.49) | 1.23 (0.44 to 3.44) | 0.903 | 234 |
| High baseline comparative risk conviction | 1.07 (0.27 to 4.28) | **4.57 (1.07 to 19.55)** | **5.82 (1.19 to 28.40)** | 1.27 (0.27 to 6.02) | **0.020** | 164 |

**Secondary continuous outcomes:** Difference (for example, Control group minus all intervention groups) and 98.75% CI estimated from ANCOVA model with adjustment for baseline and stratification variables (age, sex and risk relative to an individual with a recommended lifestyle).

p-value from a 3 degrees of freedom (d.f.) F-test of the null hypothesis, within an ANCOVA model. Null hypothesis is that there is no difference between the four randomised groups.

**Secondary categorical outcomes:** Difference in probability of outcome and 98.75% confidence interval estimated from logistic regression model, adjusted for baseline value of the outcome and stratification variables (age, sex and risk relative to an individual with a recommended lifestyle).

p-value from a Wald test (3 d.f.) of the null hypothesis that there is no difference in probability of the outcome between the four randomised groups.

**Supplementary Table S7**. Pairwise comparisons for primary and secondary outcomes at three month follow-up in those who viewed at least one lifestyle advice page

|  | **Control vs all intervention groups** | **Bar chart vs pictographs** | **Bar chart vs qualitative scale** | **Pictographs vs qualitative scale** |  |  |
| --- | --- | --- | --- | --- | --- | --- |
|  | Difference  (98.75% CI) | Difference  (98.75% CI) | Difference  (98.75% CI) | Difference  (98.75% CI) | *p* value | *n* in model |
| **Primary outcome** |  |  |  |  |  |  |
| Risk relative to individual of the same age and  sex with a recommended lifestyle (RRI) | -0.07 (-0.21 to 0.08) | -0.07 (-0.23 to 0.09) | -0.09 (-0.25 to 0.06) | -0.03 (-0.18 to 0.13) | 0.280 | 352 |
| Baseline RRI ≤ 1.5 | 0.003 (-0.11 to 0.12) | 0.01 (-0.11 to 0.13) | 0.01 (-0.12 to 0.13) | -0.01 (-0.13 to 0.11) | 0.996 | 244 |
| Baseline RRI >1.5 | -0.21 (-0.60 to 0.18) | -0.26 (-0.68 to 0.16) | -0.28 (-0.70 to 0.14) | -0.02 (-0.45 to 0.40) | 0.144 | 108 |
| Male | -0.17 (-0.58 to 0.24) | -0.26 (-0.65 to 0.13) | -0.29 (-0.66 to 0.09) | -0.02 (-0.41 to 0.36) | 0.139 | 102 |
| Female | -0.008 (-0.14 to 0.12) | 0.004 (-0.14 to 0.15) | 0.003 (-0.15 to 0.15) | -0.001 (-0.15 to 0.15) | 0.999 | 250 |
|  |  |  |  |  |  |  |
| **Secondary continuous outcomes** |  |  |  |  |  |  |
| Lifestyle |  |  |  |  |  |  |
| BMI | -0.33 (-1.40 to 0.74) | -0.72 (-1.86 to 0.41) | -0.51 (-1.65 to 0.63) | 0.22 (-0.91 to 1.35) | 0.351 | 352 |
| Alcohol | 1.07 (-0.43 to 2.57) | -0.01 (-1.60 to 1.59) | -1.24 (-2.83 to 0.35) | -1.23 (-2.81 to 0.35) | 0.042 | 346 |
| Physical activity | 0.10 (-0.74 to 0.94) | 0.07 (-0.82 to 0.95) | 0.17 (-0.73 to 1.06) | 0.10 (-0.79 to 0.99) | 0.959 | 325 |
| Fruit | -0.07 (-0.49 to 0.35) | -0.001 (-0.45 to 0.45) | 0.04 (-0.41 to 0.48) | 0.04 (-0.41 to 0.48) | 0.971 | 352 |
| Vegetables | 0.08 (-0.26 to 0.41) | -0.21 (-0.57 to 0.15) | -0.04 (-0.39 to 0.32) | 0.18 (-1.8 to 0.53) | 0.403 | 351 |
| Red meat | -0.17 (-0.66 to 0.32) | -0.30 (-0.83 to 0.22) | -0.20 (-0.73 to 0.32) | 0.10 (-0.42 to 0.62) | 0.400 | 352 |
| Processed meat | -0.08 (-0.47 to 0.31) | -0.40 (-0.82 to 0.02) | 0.02 (-0.39 to 0.44) | **0.43 (0.01 to 0.84)** | 0.038 | 352 |
|  |  |  |  |  |  |  |
| **Secondary categorical outcomes** | **OR**  **(98.75% CI)** | **OR**  **(98.75% CI)** | **OR**  **(98.75% CI)** | **OR**  **(98.75% CI)** | ***p* value** | ***n* in model** |
| Quit smoking | 0.51 (0.09 to 3.08) | 1.54 (0.27 to 8.92) | 1.68 (0.29 to 9.84) | 1.09 (0.18 to 6.70) | 0.686 | 352 |

**Primary and Secondary continuous outcomes:** Difference (for example, Control group minus all intervention groups) and 98.75% CI estimated from ANCOVA model with adjustment for baseline and stratification variables (age, sex and risk relative to an individual with a recommended lifestyle).

p-value from a 3 degrees of freedom (d.f.) F-test of the null hypothesis, within an ANCOVA model. Null hypothesis is that there is no difference between the four randomised groups.

**Secondary categorical outcomes:** Difference in odds of outcome and 98.75% confidence interval estimated from logistic regression model, adjusted for baseline value of the outcome and stratification variables (age, sex and risk relative to an individual with a recommended lifestyle).

p-value from a Wald test (3 d.f.) of the null hypothesis that there is no difference in probability of the outcome between the four randomised groups.
